# Nab-paclitaxel combined with cadonilimab (AK104) as second-line treatment for advanced gastric cancer: protocol for a phase II prospective, multicenter, single-arm clinical trial

**DOI:** 10.3389/fimmu.2025.1519545

**Published:** 2025-02-25

**Authors:** Jing Wei, Pengfei Zhang, Qiancheng Hu, Xiaolong Cheng, Chaoyong Shen, Zhixin Chen, Wen Zhuang, Yuan Yin, Bo Zhang, Hongfeng Gou, Kun Yang, Feng Bi, Ming Liu

**Affiliations:** ^1^ Gastric Cancer Center, Department of Medical Oncology, West China Hospital, Sichuan University, Chengdu, Sichuan, China; ^2^ Department of General Surgery/Gastric Cancer Center, West China Hospital, Sichuan University, Chengdu, Sichuan, China; ^3^ Division of Abdominal Cancer, Department of Medical Oncology, Cancer Center and Laboratory of Molecular Targeted Therapy in Oncology, West China Hospital, Sichuan University, Chengdu, China

**Keywords:** gastric cancer, cadonilimab (AK104), nab-paclitaxel, immunotherapy, phase II clinical trial

## Abstract

**Background:**

Gastric cancer (GC) is one of the most prevalent malignant tumors worldwide, often diagnosed at an advanced stage with a poor prognosis. Paclitaxel, nab-paclitaxel, and irinotecan, either as monotherapies or in combination with ramucirumab, are currently standard second-line treatments for GC. However, the efficacy of these therapies is limited, necessitating the development of new combination strategies to improve response rates. Immune checkpoint inhibitors (ICIs) have shown success in first-line treatment for advanced GC, leading to interest in immune rechallenge strategies for second-line treatment. Re-challenging patients with ICIs after progression on first-line treatment may restore immune responses and provide additional clinical benefit. Recently, cadonilimab (AK104), a bispecific antibody targeting PD-1 and CTLA-4, has demonstrated promising antitumor activity when combined with chemotherapy in advanced gastric and gastroesophageal junction (GEJ) adenocarcinoma. However, the efficacy and safety of nab-paclitaxel combined with AK104 for the treatment of advanced GC remain unclear. Furthermore, identifying predictive biomarkers of efficacy is essential to developing personalized treatment strategies. This study aims to explore the safety and efficacy of nab-paclitaxel combined with AK104 as a second-line treatment for patients who have progressed after first-line chemoimmunotherapy, focusing on evaluating the therapeutic effect of ICIs rechallenge in gastric cancer.

**Methods:**

This is a prospective, multicenter, open-label, single-arm Phase II clinical study. Eligible patients were histologically or cytologically diagnosed with unresectable recurrent or metastatic GC, failed first-line chemotherapy in combination with immune checkpoint inhibitor, aged between 18-75 years old, expected survival ≥3 months, and with a physical status of 0 or 1 in the Eastern Cooperative Cancer Group (ECOG). Enrolled patients will receive intravenous cadonilimab (AK104) 6 mg/kg on days 1, and 15, and intravenous nab-paclitaxel 100 mg/m^2^ every four weeks on days 1, 8, and 15. The primary endpoints were objective response rate (ORR), and secondary endpoints were disease control rate (DCR), progression-free survival (PFS), and overall survival (OS). The exploratory objective was to identify biomarkers associated with efficacy, mechanism of action, and safety. A total of 59 participants were planned to be recruited using Simon’s two-stage design. The trial was initiated in June 2024 in China.

**Discussion:**

This study is the first prospective trial to evaluate the combination of nab-paclitaxel and cadonilimab as second-line treatment after first-line chemoimmunotherapy failure. By investigating immune rechallenge, it aims to reactivate anti-tumor immune responses and improve clinical outcomes in GC patients. The exploration of predictive biomarkers, such as ctDNA, TMB, MSI, PD-L1 expression, TIL profiles, and gut microbiota, will help personalize treatment and identify patients most likely to benefit from immune rechallenge. This trial could provide valuable insights into overcoming immune resistance and contribute to developing a promising second-line therapeutic strategy for advanced GC.

**Clinical trial registration:**

ClinicalTrials.Gov, identifier NCT06349967

## 1 Introduction

Gastric cancer (GC) is the fifth most common cancer worldwide, with over 2 million new cases and over 611,720 deaths expected in 2024, ranking it as the fourth leading cause of cancer-related deaths (1). Most GC cases are diagnosed at an advanced stage, making radical surgical resection impossible. Currently, fluorouracil-based systemic chemotherapy is the primary treatment for advanced or metastatic GC. However, chemotherapy alone has limited efficacy, and treatment advancements have reached a bottleneck. Recently, immunotherapy has emerged as a promising treatment, showing effective progress in various tumor types (2–4). Immune checkpoint inhibitors (ICIs), such as PD-1 and CTLA-4 inhibitors, restore T cell function by alleviating the immunosuppressive effects of the tumor microenvironment, enabling T cells to effectively attack tumor cells. Studies, including CheckMate 649, ORIENT-16, KEYNOTE-859, GEMSTONE-303, and COMPASSION-15/AK104-302, have demonstrated that combining chemotherapy with ICIs (Nivolumab, Sintilimab, Pembrolizumab, Sugmilimab, and Cadonilimab) significantly improves overall survival (OS) and progression-free survival (PFS) compared to chemotherapy alone, facilitating the transition from first-line treatments to the broader adoption of chemoimmunotherapy in advanced GC (5–10). Despite these improvements, the 5-year survival rate for advanced or metastatic GC remains low (approximately 5-20%), and most patients experience disease progression during immunotherapy. After progression on first-line treatment, current second-line therapies include single-agent chemotherapy (paclitaxel, irinotecan, docetaxel, nab-paclitaxel) or paclitaxel combined with ramucirumab (11–14). However, response rates for these treatments are limited (approximately 10-30%), underscoring the need for new combination strategies to enhance second-line treatment efficacy for GC (15–18).

Tumor cells evade immune detection by upregulating immune checkpoint molecules such as CTLA-4 and PD-1 on T lymphocytes. In gastric adenocarcinoma, high expressions of PD-L1 (around 40%) and CTLA-4 (around 85%) are associated with poor prognosis (19–22). Dual-targeted immunotherapy, validated in both preclinical and clinical studies, has shown efficacy, with CTLA-4 and PD-1 inhibitors working synergistically to restore T cell function through distinct mechanisms (23–27). CTLA-4 upregulation during T cell activation suppresses T cell activity, particularly on tumor-infiltrating regulatory T cells (Tregs) and exhausted effector T cells (Teffs). Blockade of CTLA-4 enhances T cell activation and reduces Treg infiltration, alleviating the immunosuppressive tumor microenvironment. Similarly, PD-1 upregulation following T cell activation suppresses T cell responses by binding its ligand, and inhibiting PD-1/PD-L1 signaling can reinvigorate tumor-reactive T cells. However, recent studies suggest that PD-L1 blockade may paradoxically promote Treg activity, leading to therapeutic resistance, which can be reversed by depleting Tregs (28). Additionally, context-dependent PD-(L)1 checkpoint activation induced by CTLA4-Ig therapy may further suppress T cell activity (29). These findings highlight the potential synergy between checkpoint inhibitors and combination therapies. Chemotherapy also enhances immune responses by inducing tumor apoptosis, upregulating MHC-I, promoting dendritic cell maturation, and inhibiting immunosuppressive cells like Tregs, MDSCs, and TAMs. Combining ICIs with chemotherapy has shown synergistic anti-tumor effects, supporting the rationale for combining chemotherapy with dual immune checkpoint blockade (anti-CTLA-4 and anti-PD-1) in solid tumors (30, 31).

Nab-paclitaxel, a novel formulation of paclitaxel, improves drug concentration and uptake in tumor tissues compared to traditional solvent-based paclitaxel. The ABSOLUTE study showed that nab-paclitaxel is non-inferior to weekly solvent-based paclitaxel and has been recommended as a standard second-line treatment for GC (32). However, its efficacy as a single-agent second-line treatment remains limited, highlighting the need for novel strategies to improve clinical outcomes.

Cadonilimab (AK104) is a bispecific antibody that targets both the PD-1 and CTLA-4 immune checkpoint pathways, utilizing a 4-valent IgG1-ScFv format. It reverses T-cell depletion by facilitating the endocytosis of cell-surface PD-1 and CTLA-4 receptors, which subsequently induces the secretion of IL-2 and IFN-γ. This mechanism not only reduces immune-related adverse events (irAEs) but also enhances the ability of T cells to kill tumor cells (33). Cadonilimab is currently approved in China for treating recurrent or metastatic cervical cancer that has progressed during or after platinum-based chemotherapy (34). In the multicenter, open-label Phase 1b/2 COMPASSION-03 trial, cadonilimab demonstrated significant antitumor activity and a manageable safety profile in patients with advanced solid tumors (9, 23, 35, 36). More recently, in the Phase 3 COMPASSION-15/AK104-302 trial, cadonilimab combined with chemotherapy as a first-line treatment for patients with HER2-negative unresectable advanced or metastatic gastric or gastroesophageal junction (GEJ) adenocarcinoma showed an objective remission rate (ORR) of up to 65.2% and an overall survival (OS) of up to 15 months (37). Additionally, this combination therapy reduced the risk of death by 44% in patients with high PD-L1 expression (CPS ≥ 5) and by 30% in those with low PD-L1 expression (CPS < 5).

This study aims to evaluate the efficacy and safety of nab-paclitaxel combined with cadonilimab as a second-line treatment for advanced GC following the failure of first-line chemoimmunotherapy (PD1 inhibitors). Additionally, emerging evidence highlights the critical role of gut microbiota and predictive biomarkers in shaping ICI responses, but their integration into GC treatment remains limited. This study will explore diagnostic biomarkers and gut microbiota as predictive and prognostic factors, providing deeper insights into the mechanisms of treatment resistance and therapeutic efficacy. By combining these exploratory analyses with efficacy assessments, the study represents a significant advancement toward personalized immunotherapy, addressing unmet clinical needs in the management of GC.

## 2 Methods and analysis

### 2.1 Study design

**Figure 1:**
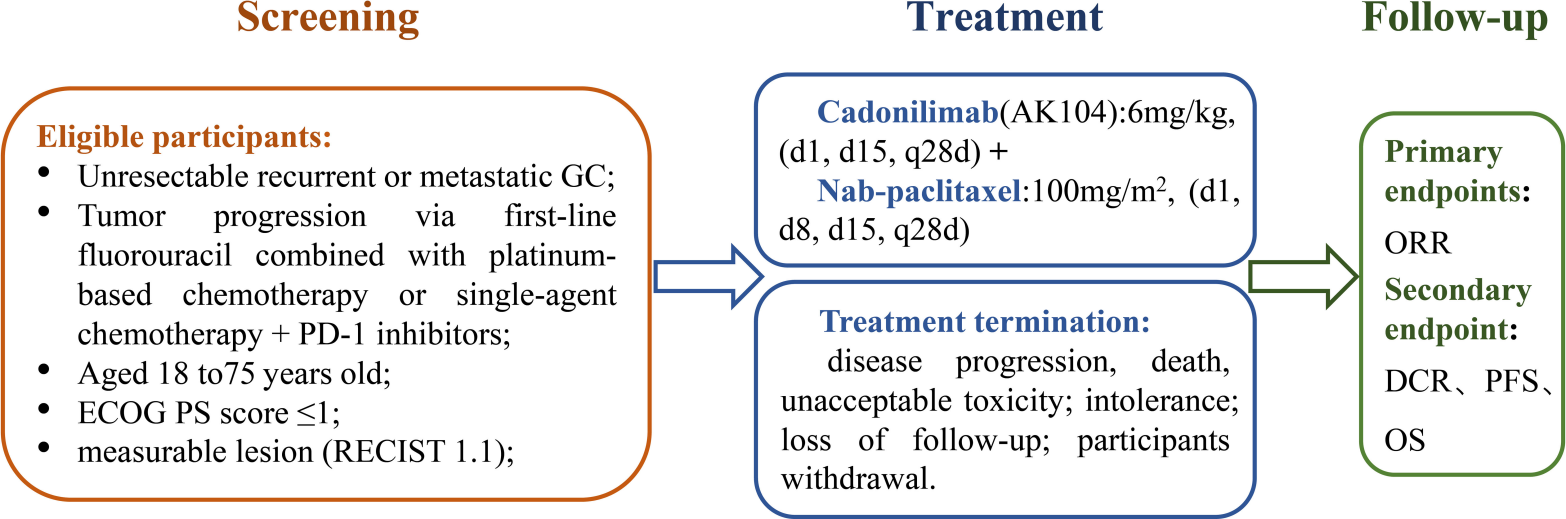
Study design of the clinical trial evaluating nab-paclitaxel combined with cadonilimab.

This prospective, multicenter, open-label, single-arm phase II clinical study aims to evaluate the efficacy and safety of nab-paclitaxel in combination with cadonilimab (AK104) as a second-line treatment for patients with gastric cancer (GC) who have failed first-line fluorouracil-based or platinum-based combination immunotherapy. The study design is illustrated in **Figure 1**.

Eligible patients are those with histologically or cytologically confirmed unresectable, recurrent, or metastatic GC who have experienced disease progression following first-line chemotherapy combined with immune checkpoint inhibitors (PD-1/PD-L1). Patients enrolled in the study will receive nab-paclitaxel in combination with cadonilimab until either disease progression (PD) or the onset of treatment intolerance.

The study will prospectively collect data on overall response rate (ORR), disease control rate (DCR), progression-free survival (PFS), overall survival (OS), and quality of life (QoL), alongside a comprehensive assessment of the medication’s safety profile.

### 2.2 Inclusion and exclusion criteria

The inclusion and exclusion criteria are detailed in **Table 1**.

**Table 1:** Inclusion and exclusion criteria.

Primary Inclusion Criteria	Primary Exclusion Criteria
(1) Patients with unresectable gastric or gastroesophageal junction adenocarcinoma confirmed by laparoscopic exploration and pathological/cytological examination;	(1) Participation in any clinical trials of a drug, or ongoing clinical trials of other drugs within 1 month prior to enrollment;
(2) Aged 18 to 75 years;	(2) Hyper-progression occurred during first-line immunotherapy: • An increase in tumor load of more than 50% compared to the baseline period when first assessed after 2 to 4 cycles of first-line therapy; • The tumor growth rate after immunotherapy is more than twice the previous rate;
(3) Tumor progression after first-line treatment with a fluoropyrimidine (5-FU, S-1, or capecitabine) combined with a platinum-based agent (oxaliplatin or cisplatin), or single-agent chemotherapy plus a PD-1/PD-L1 inhibitor	(3) Presence of any active autoimmune disease or history of autoimmune disease (including but not limited to: interstitial pneumonia, uveitis, enteritis, hepatitis, nephritis, hyperthyroidism, hypothyroidism);
(4) Expected survival of at least 3 months;	(4) First-line immunotherapy-related Grade 3-4 immune hepatitis, immune pneumonia, and immune myocarditis, etc.;
(5) Eastern Cooperative Oncology Group (ECOG) Performance Status ≤1	(5) Currently receiving immunosuppressive agents or hormone therapy (administered systemically or locally) for the purpose of immunosuppression and having continued such treatment within two weeks prior to enrollment;
(6) Presence of at least one measurable lesion as defined by the RECIST 1.1 criteria;	(6) ≥ grade 3 bleeding event within 4 weeks prior to enrollment; thromboembolic or arteriovenous event such as cerebrovascular event (including transient ischemic attack), deep vein thrombosis, or pulmonary embolism within 6 months prior to enrollment, etc.;
(7) Patients must have adequate liver, kidney, and bone marrow function, as demonstrated by the following laboratory test criteria: • Total bilirubin ≤ 1.5 times the upper limit of normal (ULN) • Serum alanine aminotransferase (ALT) and aspartate aminotransferase (AST) levels ≤ 3 times the ULN; • Alkaline phosphatase ≤ 2.5 times the ULN (≤ 3 times the ULN if the tumor has intrahepatic invasion); • Blood creatinine ≤ 1.5 times the ULN and creatinine clearance (Ccr) ≥ 60 mL/min; • Serum amylase and lipase levels ≤ 1.5 times the ULN; • International Normalized Ratio (INR) and/or Partial Thromboplastin Time (PTT) ≤ 1.5 times the ULN; • Have not received a blood cell-boosting intervention, such as a transfusion or stimulating factor, for at least 2 weeks prior to administration, with a platelet count ≥ 75,000/mm³, hemoglobin ≥ 9 g/dL, and neutrophil count ≥ 1,500/mm³;	(7) Active or clinically significant cardiovascular disease: • Congestive heart failure: New York Heart Association (NYHA) Class > II; • Active coronary artery disease; • Arrhythmias requiring treatment other than β-blockers or digoxin; • Unstable angina (angina symptoms at rest), new angina within 3 months prior to enrollment, or new myocardial infarction within 6 months prior to enrollment;
(8) Strict contraception;	(8) Symptomatic brain metastases or meningioma;
(9) Voluntary participation and signed informed consent;	(9) Patients with other significant medical or surgical conditions that, in the investigator’s judgment, render them unsuitable for participation in this clinical trial: • Active, symptomatic interstitial lung disease, pleural effusion, or ascites causing dyspnea (grade ≥ 2); • Patients with renal failure who require hemodialysis or peritoneal dialysis; • Presence of gastrointestinal perforation, gastrointestinal obstruction, or uncontrolled diarrhea within 6 months prior to enrollment; • Presence of unhealed wounds, ulcers, or fractures;
	(10) • Must not have untreated or concurrent other tumors, except for treated cervical carcinoma in situ, basal cell carcinoma, or superficial bladder tumors; • Enrollment is allowed if the tumor has been eradicated and there is no evidence of disease for more than 3 years; • Treatment of all other tumors must have been completed at least 3 years prior to enrollment;
	(11) Patients with a history of HIV infection or active hepatitis B or C;
	(12) Ongoing infection with severity greater than grade 2;
	(13) Pregnant or breastfeeding females;
	(14) Substance abuse, along with medical, psychological, or social conditions, may impact patient enrollment and the evaluation of experimental results;
	(15) • During the treatment period, no additional antineoplastic therapies (chemotherapy, radiotherapy, surgery, immunotherapy, biological therapy, or chemoembolization) beyond the investigational drug are permitted; • Palliative external-beam radiotherapy to non-target lesions is allowed;
	(16) Has previously received the same class of chemotherapeutic agents or immune checkpoint inhibitors;
	(17) • Allergy or suspected allergy to the study drug or any drug of the same class; • Major surgery, open biopsy, or major traumatic surgery within 4 weeks prior to enrollment; • History of organ transplantation (including corneal transplantation); History of allogeneic blood transfusion within the past 6 months; • Vaccination history within 4 weeks prior to enrollment;
	(18) Patients deemed unsuitable for inclusion in this study at the discretion of the investigator.

### 2.3 Treatment

Cadonilimab (AK104): Administered at a dose of 6 mg/kg via intravenous infusion over 30-60 minutes on Days 1 and 15 of each 28-day cycle (q28d).

Nab-paclitaxel: Administered at a dose of 100 mg/m² via intravenous infusion over 30-40 minutes, initiated 30 minutes after the completion of the cadonilimab (AK104) infusion, on Days 1, 8, and 15 of each 28-day cycle (q28d).

Eligible participants will receive nab-paclitaxel in combination with cadonilimab until one of the following occurs: PD, death, loss to follow-up, unacceptable toxicity, withdrawal of informed consent, or other treatment termination criteria as specified in the study protocol.

### 2.4 Objectives and endpoints

#### 2.4.1 Objectives

##### 2.4.1.1 Primary purpose

1. To evaluate the ORR of nab-paclitaxel in combination with cadonilimab (AK104) as a second-line treatment for GC patients who have failed first-line fluorouracil-based or platinum-based combination immunotherapy.

##### 2.4.1.2 Secondary purpose

1. To assess the efficacy of nab-paclitaxel combined with cadonilimab (AK104) in the second-line treatment of GC patients who have failed first-line fluorouracil-based or platinum-based combination immunotherapy, including DCR, PFS, and OS.
2. To evaluate the safety, tolerability, and impact on patient QoL of nab-paclitaxel in combination with cadonilimab (AK104).

##### 2.4.1.3 Exploratory purpose

1. To investigate the antitumor efficacy, mechanism of action, and potential resistance mechanisms of nab-paclitaxel combined with cadonilimab (AK104).
2. To explore the role of tumor tissue PD-L1 expression, blood immune cell subpopulations, and serum cytokine levels as potential predictors of treatment efficacy.
3. To examine the role of liquid biopsy biomarkers, such as circulating tumor DNA (ctDNA), cell-free DNA (cf-DNA), and blood tumor mutational burden (bTMB), as predictors of efficacy, with a focus on their capacity to monitor minimal residual disease or treatment response.
4. To explore the association between gut microbiota composition and immunotherapy response using large-scale metagenomic analysis, aiming to identify microbial signatures that correlate with treatment outcomes.
5. To assess the impact of the treatment protocols on patient QoL using the QLQ-LC13 and EORTC QLQ-C30 questionnaires, with an emphasis on patient-reported outcomes related to physical and emotional well-being.

#### 2.4.2 Endpoints

##### 2.4.2.1 Primary endpoint

Objective response rate (ORR): defined as the proportion of subjects whose tumors achieved complete response (CR) or partial response (PR) after treatment. Evaluated by the Blinded Independent Image Review Committee (BIIRC). This serves as the primary indicator of treatment efficacy.

##### 2.4.2.2 Secondary endpoints

Disease Control Rate (DCR): defined as the proportion of subjects whose tumors achieved CR, PR, or stable disease (SD) after treatment. This reflects broader disease stabilization benefits.

Progression-free survival (PFS): defined as the time from randomization to tumor progression or death due to any cause, providing insights into treatment durability.

Overall survival (OS): defined as the time from randomization to death due to any cause (last follow-up for patients lost to follow-up; end of follow-up date for patients alive at the end of the study). This serves as a critical measure of long-term efficacy.

##### 2.4.2.3 Exploratory endpoint

Accompanying diagnostic biomarkers and gut flora characterization: This exploratory analysis aims to identify predictive and prognostic biomarkers, with a particular focus on gut microbiota and its interaction with immunotherapy. These findings may provide novel insights into the mechanisms underlying treatment responses and immune-related adverse events (irAEs).

### 2.5 Efficacy and safety assessment

#### 2.5.1 Efficacy assessment

Laboratory tests, including hematology, liver and kidney function, electrolytes, cardiac markers, thyroid function, coagulation, and transmission nine tests, were performed on the first day of each treatment cycle. Tumor biomarker assessments, including carbohydrate antigen 19-9 (CA19-9), CA125, carcinoembryonic antigen (CEA), and alpha-fetoprotein (AFP), were also conducted. Additionally, 12-lead electrocardiograms and echocardiograms were performed at the same time. These tests were performed according to the protocol, with additional assessments performed if clinically indicated.

Antitumor efficacy was evaluated every 8 weeks (approximately at the end of every two dosing cycles) via CT or MRI examinations (chest, abdomen, pelvis, and any other site suspected of having tumor lesions). Tumor imaging response was assessed according to RECIST version 1.1. Tumor assessments may be conducted more frequently if clinically necessary, based on the investigator’s judgment.

All procedures were conducted in accordance with the study protocol and with informed consent from the participants.

#### 2.5.2 Safety evaluation

Safety assessments will be conducted throughout the study, with participants closely monitored for adverse events (AEs) until resolution, stabilization, or confirmation of non-clinical significance.

AE Definition: defined as any untoward medical occurrence in a participant receiving the investigational product, irrespective of its causal relationship to the treatment. AEs will be evaluated for severity (graded per NCI-CTCAE v5.0), duration, and relationship to the study treatment.

Serious Adverse Event (SAE) Definition: defined as any event that results in death, is life-threatening, causes significant disability, requires hospitalization or prolongs an existing hospitalization, leads to congenital anomalies, or constitutes other medically significant conditions. SAEs must be reported to the regulatory authority, ethics committee, and sponsor within 24 hours, with follow-up reports submitted as needed. All AEs/SAEs must be thoroughly documented in the case report form (CRF), including details on onset, duration, resolution, and any interventions taken.

The study team will follow the study protocol and standard operating procedures (SOPs) for AE/SAE management, implementing necessary measures such as dose adjustments or treatment discontinuation when required.

### 2.6 Management plan for adverse events

#### 2.6.1 Management of adverse events related to AK104

For low-grade adverse events (Grade 1 or 2), symptomatic and topical treatments are recommended. Persistent low-grade events or severe events (Grade ≥3) should be managed with systemic corticosteroids, such as prednisone or intravenous equivalents.

Discontinuation of AK104 therapy is not mandatory for Grade 3 or 4 inflammatory responses (e.g., inflammation at metastatic sites or lymph nodes) attributable to a localized tumor response. In cases of multiple concurrent low-grade AEs that individually would not necessitate therapy termination, the decision to discontinue AK104 treatment will be at the investigator’s discretion.

The investigator will evaluate the severity of immune-related adverse events (irAEs) using the NCI CTCAE version 5.0 grading criteria and adjust AK104 therapy as necessary. General management recommendations for irAEs are outlined in **Table 2**.

**Table 2:** General principles for dose adjustment.

Grade of irAE	Treatment plan adjustments
Grade 1 irAE	No adjustments to the treatment plan are required
Grade 2 irAE	Suspend AK104 therapy until grade 2 adverse events subside to ≤ grade 1 or baseline level: a) If toxicity worsens, it is recommended to manage as grade 3 or 4; b) If toxicity improves, consider resuming AK104 therapy at the appropriate scheduled treatment visit; c) Permanent discontinuation of AK104 should be considered if grade 2 irAE does not subside to ≤ grade 1 or baseline level within 12 weeks after symptomatic treatment;
Grade 3 irAE	Suspend AK104 therapy until grade 3 adverse events (irAE) regress to ≤ grade 1 or baseline level: a) If toxicity improves, the investigator may consider whether to permanently terminate AK104 treatment based on the type of individual toxicity, its occurrence, and the course of regression; b) Permanent termination of AK104 therapy is recommended if grade 3 irAE does not regress to ≤ grade 1 or baseline level within 12 weeks with symptomatic treatment;
Grade 4 irAE	The treatment with AK104 must be discontinued permanently;

### 2.7 Study follow-up

Overall survival (OS) analysis will be conducted throughout the trial period. During the follow-up phase, subjects will be assessed every three months to document their survival status, including PD, AEs, and QoL where applicable. Follow-up visits may be conducted through in-person consultations, phone calls, or electronic surveys, depending on patient circumstances.

Follow-up will continue until the subject’s death, loss to follow-up, or the end of the study period. All data collected during follow-up will be systematically recorded in the study database. Measures will be implemented to ensure adherence to follow-up schedules, including regular reminders and patient support initiatives.

### 2.8 Exploratory endpoint analysis

This study focuses on biomarker analysis and gut microbiota characterization to explore the predictive value of peripheral biomarkers in assessing disease activity and survival benefits associated with AK104 treatment, leveraging advancements in liquid biopsy technologies and their therapeutic potential.

Peripheral blood samples were collected before the first dose (Day 1, Cycle 1) and at disease progression. Fresh whole blood was processed within 2 hours to extract plasma, which was stored at -80°C for next-generation sequencing (NGS) analysis. NGS was used to evaluate cancer-related genes, including ctDNA, cfDNA somatic mutations, and bTMB. Changes in immune cell subsets, cytokines, and tumor immunotherapy biomarkers were also assessed.

Fecal samples, collected before treatment initiation, were analyzed using large-scale metagenomic sequencing. This approach provided comprehensive taxonomic and functional profiling to assess associations between gut microbiota composition and immunotherapy response.

Data from biomarker and microbiota analyses were evaluated using multivariate regression and survival models, with adjustments for confounding factors. All patients provided informed consent, and the study protocol received approval from the institutional review board (IRB).

### 2.9 Sample size calculation and statistical analysis

Sample size calculations were performed using Simon’s optimal two-stage design to balance ethical considerations and resource efficiency. This design minimizes the expected sample size under the null hypothesis while ensuring adequate power to detect a meaningful treatment effect if the alternative hypothesis is true. Simon’s two-stage design was chosen due to its widespread use in early-phase clinical trials to assess efficacy and safety efficiently.

The minimax design parameters were set as follows: an alpha error of 5% and a power (1-β) of 80% were used for calculations. The minimax two-stage design resulted in parameters (6/31, 15/53). Stage 1: Enroll 31 patients. If ≤6 patients achieve ORR, the trial is terminated due to futility. Stage 2: If >6 responses are observed, an additional 22 patients are enrolled, resulting in a total of 53 patients. Success Criteria: The treatment regimen is considered successful if ≥15 patients achieve ORR. To account for potential patient dropout, the sample size was increased by 10%, resulting in a final planned enrollment of 59 patients. PFS and OS will be analyzed using the Kaplan-Meier method. Median survival times and 95% confidence intervals (CI) will be reported. Differences between groups will be assessed using the log-rank test. If applicable, Cox proportional hazards regression analysis will be performed to evaluate the impact of baseline covariates on survival outcomes. ORR, DCR, and AEs will be analyzed using descriptive statistics. The Clopper-Pearson method will be used to calculate 95% confidence intervals for proportions. Subgroup analyses may be conducted based on predefined stratification factors. Sample size calculations were performed using PASS 2024 software (version 24.0.2, NCSS).

Exploratory analyses will be conducted to identify predictive and prognostic biomarkers using advanced immunoassays and bioinformatics tools. Adverse events will be graded according to CTCAE v5.0, and comparisons between different severity grades will be performed using chi-square or Fisher’s exact tests. This statistical approach ensures rigorous evaluation of efficacy and safety while accommodating the exploratory nature of biomarker identification.

## 3 Discussion

Although first-line chemotherapy regimens for advanced gastric cancer (GC) have improved, the survival times remains below 12 months, highlighting the need for further efficacy advancements. Immunotherapy has made significant strides in treating advanced GC, overcoming the long-standing survival limitations associated with traditional chemotherapy (38–40). Notably, the CheckMate-649 trial, a randomized, open-label, multicenter Phase III study, evaluated nivolumab combined with chemotherapy, nivolumab plus ipilimumab, and single-agent chemotherapy in HER2-negative advanced GC patients. Results showed that nivolumab plus chemotherapy significantly improved OS and PFS compared to chemotherapy alone, with an acceptable safety profile (41). Importantly, in patients with PD-L1 CPS ≥ 5, nivolumab in combination with chemotherapy reduced the risk of death by 29% compared to chemotherapy alone. As a result, nivolumab combined with chemotherapy has become a recommended first-line treatment option in clinical guidelines from CSCO, NCCN, and ESMO. Similarly, the ATTRACTION-04 trial, a randomized multicenter Phase II/III study, demonstrated that first-line nivolumab with chemotherapy significantly improved PFS in Asian patients with HER2-negative advanced or recurrent GC (PFS: 10-45 months vs. 8-34 months; HR 0.68; 98.51% CI: 0.51-0.90; P=0.0007), suggesting it may become the new standard of care (42). Additional studies, including ORIENT-16, RATIONALE-305, KEYNOTE-859, GEMSTONE-303, and COMPASSION-15/AK104-302, showed that combining chemotherapy with PD-1/PD-L1 monoclonal antibodies such as sindilizumab, tirilizumab, pabolizumab, sugemalimab, and cadonilimab improved median overall survival (mOS) to 13-15 months. In patients with high PD-L1 expression, mOS reached 15-18 months, with an ORR of approximately 60%.

The combination of ICIs and chemotherapy has now become the standard first-line treatment for advanced GC without driver gene mutations, including in unresectable locally advanced GC and perioperative settings (43–45). Despite these significant improvements in prognosis, around 40% of patients still experience PD, and OS often falls short of expectations. Re-challenge with ICIs, which involves reintroducing these agents after PD or serious irAEs, has been extensively studied in melanoma and urologic cancers (46–48). ICI rechallenge works by either reactivating the normal immune cycle or bypassing immune dysfunction to resensitize tumor cells to ICIs (49–51). Tumor cells that initially responded to ICIs may still harbor susceptible populations, and the interactions between the immune system and the tumor microenvironment may provide new opportunities for anti-tumor responses. Additionally, immunotherapy can alter the tumor neoantigen profile, enabling tumors to evade recognition by memory T cells, but ICI rechallenge can restore T cells’ ability to recognize these neoantigens (4, 52). Studies such as CheckMate 066/067 and KEYNOTE-010 have demonstrated that rechallenge with nivolumab and pembrolizumab resulted in lesion shrinkage and significant OS improvements in patients with advanced melanoma and PD-L1-positive advanced non-small-cell lung cancer (NSCLC), with no additional AEs reported (53–56).

Although chemotherapy combined with immunotherapy demonstrates synergistic effects through the promotion of immunogenic cell death (ICD) and the release of neoantigens, the rechallenge of ICIs in advanced GC remains underexplored due to limited clinical evidence and the lack of large-scale prospective studies. Furthermore, identifying biomarkers for ICI rechallenge in advanced GC is crucial for facilitating precise, individualized immunotherapy and improving the effectiveness of combination therapies. Significant progress has been made in identifying biomarkers for gastrointestinal cancers, with PD-L1 expression, microsatellite instability (MSI), and mismatch repair deficiency (dMMR) emerging as key indicators (57). Ongoing research is also focusing on other biomarkers, including tumor mutational burden (TMB), circulating tumor DNA (ctDNA), Epstein-Barr virus (EBV), and the gut microbiome. Advanced immunoassays, multi-omics approaches, and bioinformatics tools offer additional potential for identifying biomarkers predictive of positive responses to ICI rechallenge. By integrating these biomarkers with clinicopathological features, we can more effectively stratify advanced GC patients undergoing ICI treatment, explore mechanisms of ICI drug resistance, identify potential strategies for sensitization, and ultimately improving overall prognosis (58–62).

Managing irAEs is a critical aspect of immunotherapy. As a bispecific antibody targeting both PD-1 and CTLA-4, cadonilimab may have a unique irAE profile. Common irAEs include dermatologic, gastrointestinal, hepatic, and endocrine disorders, all of which require timely diagnosis and management. Strategies such as early corticosteroid intervention, immune-modulating agents, and careful monitoring are essential to mitigate irAEs. Our study emphasizes the need for standardized irAE management protocols, especially for novel agents like cadonilimab.

This study has several strengths, including the investigation of a novel combination therapy, exploration of predictive biomarkers, and well-defined patient stratification. However, it also has limitations, as it was conducted within a single geographic region and focused primarily on an Asian population. While the findings offer valuable insights into the efficacy of cadonilimab in this group, their generalizability to other populations remains uncertain. Gastric cancer epidemiology, genetic diversity, and healthcare access vary globally, which may influence treatment responses and outcomes. Additionally, potential confounding from prior immunotherapy treatment could impact the interpretation of results. Future multicenter and global studies are needed to validate these results and ensure the broader applicability of cadonilimab-based regimens.

This Phase II clinical trial is the first to evaluate nab-paclitaxel combined with cadonilimab (AK104) as a second-line treatment for advanced GC. The study aims to investigate the efficacy and safety of ICI rechallenge in patients who have progressed after first-line chemoimmunotherapy, focusing on immune resistance mechanisms and predictive biomarkers of susceptibility. By exploring real-world rechallenge strategies, this research provides evidence-based insights to optimize immunotherapy outcomes and paves the way for future studies, highlighting cadonilimab’s potential to transform treatment for advanced gastric cancer.
